# Multi-trophic native and non-native prey naïveté shape marine invasion success

**DOI:** 10.1371/journal.pone.0221969

**Published:** 2019-09-06

**Authors:** Katherine J. Papacostas, Amy L. Freestone

**Affiliations:** Department of Biology, Temple University, Philadelphia, PA, United States of America; Uppsala Universitet, SWEDEN

## Abstract

Invasive predators have caused rapid declines in many native prey species across the globe. Predator invasion success may be attributed to prey naïveté, or the absence of anti-predator behavior between native and non-native species. An understanding of the effects of naïveté at different timescales since introduction and across multiple trophic levels is lacking, however, particularly in marine systems. Given the central role of trophic interactions in invasion dynamics, this knowledge gap limits the ability to predict high impact predator invasions. Naïveté was examined across three trophic levels of marine invertebrates: a native basal prey (hard clam), two non-native intermediate predators (the recently-introduced Asian shore crab and the long-established European green crab), a native intermediate predator (juvenile blue crabs), and a native top predator (adult blue crab). We hypothesized that naïveté would be more pronounced in trophic interactions involving the recently-introduced non-native predator in comparison to the long-established non-native and native intermediate predators. We further hypothesized that the recently-introduced intermediate predator would both benefit from naïveté of the native basal prey and be hindered by higher mortality through its own naïveté to the native top predator. To test these hypotheses, three laboratory experiments and a field experiment were used. Consistent with our hypotheses, basal prey naïveté was most pronounced with the recently-introduced intermediate predator, and this increased the predator’s foraging success. This recently-introduced intermediate predator, however, exhibited an ineffective anti-predator response to the native top predator, and was also preyed upon more in the field than its long-established and native counterparts. Therefore, despite direct benefits from basal prey naïveté, the recently-introduced intermediate predator’s naïveté to its own predators may limit its invasion success. These results highlight the importance of a multi-trophic perspective on predator-prey dynamics to more fully understand the consequences of naïveté in invasion biology.

## Introduction

Predator-prey interactions are often attributed to coevolution [1, 2], but a lack of shared evolutionary history between native and non-native species (i.e. naïveté) alters these interactions substantially and can contribute to the success of non-native species [3]. Prey naïveté can occur when a prey species lacks shared evolutionary history with a predator [4] and can influence the lag time and spread rate of an invasion [1, 3, 5]. Depending on a species’ experience with a particular predator or related predators, four different types of naïveté can emerge [6, 7]: prey can fail to recognize a non-native predator, recognize a predator but respond ineffectively, exhibit an effective anti-predator response that is unsuccessful due to superior predator tactics, or over-respond and experience sublethal costs of predation (e.g. reducing foraging time to hide from a predator). A reduction in naïveté can evolve over several generations [8] or can be achieved via learning within a generation [9]. Therefore, although naïveté may initially benefit non-native predator populations, strong selection pressure on prey populations may lead to altered behavioral responses and coexistence with the non-native species.

Within the past fifteen years, studies on prey naïveté have expanded rapidly, particularly in terrestrial and freshwater systems, providing new perspectives [1, 3, 6, 7, 10] that are being incorporated into recent invasion biology frameworks [11]. There has been far less empirical attention, however, given to naïveté in marine systems. This gap in the literature may be due to limited data on marine predator invasions [12] or the idea that the increased connectivity in marine systems compared to terrestrial and freshwater systems make marine prey naïveté much less likely [6, 13]. This inference about limited naïveté in marine environments, however, is largely based on pelagic systems ([6], although see [13] for an estuarine example). Existing marine studies conducted in coastal systems have indeed shown that naïveté can occur (e.g. [4, 14]) and can have significant impacts on coastal communities (e.g. [15]).

As non-native predators are often prey themselves, they also face predation risk from higher trophic levels and could be at a disadvantage due to their own naïveté [2]. While naïveté can manifest when non-native predators consume naïve native prey resources, potentially facilitating establishment (a resource effect), non-native prey could also be naïve to native predators, which could limit establishment (a predator effect). This multi-trophic naïveté likely occurs in ecological communities, but has not been examined rigorously in either terrestrial or aquatic systems [1]. Past studies have primarily examined two trophic levels, either a naïve non-native prey and native predator or a naïve native prey and non-native predator (e.g. [2, 4]). While such studies are extremely valuable, a clear understanding of how these findings extrapolate to a multi-trophic system is lacking.

The aim of this study was to examine naïveté at different timescales since introduction and across multiple trophic levels. We used four marine invertebrates representing three trophic levels as model taxa: a native basal prey, two non-native predators (one recently-introduced, one long-established), one native intermediate predator, and a native top predator. We hypothesized that naïveté would be more pronounced in trophic interactions involving the recently-introduced non-native predator in comparison to the long-established non-native and native intermediate predators. We further hypothesized that the recently-introduced intermediate predator would both benefit from naïveté of the native basal prey (resource effect) and be hindered by higher mortality through its own naïveté to the native top predator (predator effect).

## Materials and methods

### Ethics statement

Permission was granted from the Rutgers University Marine Field Station for use of field sites.

### Study system

We used a tri-trophic marine invertebrate system to test hypotheses about the potential for prey naïveté at different stages of invasion and across trophic levels. This tri-trophic system included a native basal prey, two non-native intermediate predators, one native intermediate predator, and a native top predator, in a temperate estuary of the Western Atlantic Ocean (New Jersey [NJ], USA). The two non-native intermediate predators differed in time since introduction; the recently-introduced Asian shore crab, *Hemigrapsus sanguineus* [16], was first sighted in the study area 25–30 years ago [17], and the long-established European green crab, *Carcinus maenas*, was first documented in NJ in 1817 [18] and has had a stable range along the US east coast for over a century [19]. Juveniles of the blue crab *Callinectes sapidus* served as a native intermediate predator. Adult *C*. *sapidus* served as the native top predator since they readily consume all three intermediate crab species [20], including juveniles of their own species [21], and are known to be important structuring agents in these communities [18, 22]. The native basal prey was the hard clam, *Mercenaria mercenaria*, which is an important shared prey resource of the three crabs [23–25] and burrows in the presence of predator chemical cues [26]. This tri-trophic system allowed for a temporal comparison of anti-predator behaviors among native (juvenile *C*. *sapidus*), recently-introduced non-native (*H*. *sanguineus*) and long-established non-native (*C*. *maenas*) intermediate predators, as well as a comparison of resource and predation effects on the recently-introduced *H*. *sanguineus*.

We used three laboratory experiments and a field experiment to test for naïveté across three trophic levels. We collected crab individuals both by hand and through trapping in Little Egg Harbor (39.5°N, 74.3°W) and Barnegat Bay, NJ, USA (39.9°N, 74.1°W), and clams were supplied by local hatcheries (see Acknowledgments). In the laboratory experiments, juvenile clams *M*. *mercenaria* were on average 10.8mm wide (± 2.7 SD), a size class that all three crab species are able to crush [23, 27, 28]. In the laboratory experiments, size classes of crabs used were representative of local populations: mean carapace widths were 25.9 ± 3.1mm for *H*. *sanguineus*, 57.7 ± 8.1mm for *C*. *maenas*, 68.5 ± 14.8mm for *C*. *sapidus* juveniles, and 128 ± 13.35mm for *C*. *sapidus* adults. For laboratory experiments, animals were maintained in filtered, aerated aquaria. Clams were fed regularly with cultured phytoplankton. Crabs were fed a mixed diet of clams and mussels (collected in Little Egg Harbor) as well as algae, except for those that were used in experiments with live clams, which were instead fed a purely algal diet to remove any bivalve chemical cues [29]. Crabs were not fed for a period of 48 hours prior to experiments to standardize hunger levels. All laboratory experiments were conducted at ~23°C, which is well within the range of summer water temperatures in southern NJ [30], and each trial used fresh artificial seawater (salinity 30ppt) and clean aquaria to ensure the lack of external scents. For the field experiment, crabs were collected locally and used on the same day as collection. Since the three species vary in the average size of adults [31–33], the field experiment was standardized by collecting individuals of each species and matching them by weight (21.65 ± 13.15g).

### Native basal prey naïveté: *M*. *mercenaria* burrowing experiment

To test the hypothesis that our basal prey would have more pronounced naïveté, and thus weaker defense behavior (shallower burrowing), in response to the recently-introduced intermediate predator in comparison to the native or long-established intermediate predators, we ran a laboratory experiment in summer 2012. Basal prey clams, *M*. *mercenaria*, were introduced into aerated cylindrical mesocosms (30cm diameter, 37 cm depth) of sieved locally-collected sediment (15 cm depth) and artificial seawater, and given 24 hours to acclimate, with one clam per mesocosm. After acclimation, clams were exposed to one of four treatments with 10 replicates per treatment: 1) no predator, 2) one *H*. *sanguineus* individual, 3) one *C*. *maenas* individual, or 4) one juvenile *C*. *sapidus* individual. Crabs in predator treatments were caged to prevent consumption of clams. Trials ran for one week during which burrowing depth was recorded every other day. We conducted 5 trials, with each treatment replicated two times in each trial. The results were analyzed with a mixed model ANOVA in JMP Statistical Software using the REML method, with treatment as a fixed effect, trial as a random effect, and replicate (individual clam) as a random effect.

### Foraging benefits to recently-introduced intermediate predator from naïve native prey: *H*. *sanguineus* foraging experiment

We used a second laboratory experiment in summer 2013 to test the hypothesis that *M*. *mercenaria* naïveté would benefit *H*. *sanguineus* foraging success. *M*. *mercenaria* were placed in 20-gallon tanks (base dimensions 76cm × 32cm) at densities of seven clams per tank, which was within the range of natural densities of hard clams in Little Egg Harbor and Barnegat Bay, NJ [28]. Experimental tanks were aerated and contained 12cm of water (5cm above the 7cm layer of sieved sediment). *M*. *mercenaria* were placed in tanks the night before trials to allow them to acclimate. They were then exposed to *H*. *sanguineus* predation for a 24 hour period, with one crab per mesocosm. Two experimental treatments were used that enabled *H*. *sanguineus* to forage on *M*. *mercenaria* that were either 1) allowed to burrow naturally (shallower, naïve depth) or 2) placed at an experienced (deep) burrowing depth of 2.3cm. Experienced depth was determined from the 2012 burrowing experiments (*C*. *sapidus* treatment), and preliminary trials (n = 10) confirmed that *M*. *mercenaria* did not move substantially over the 24 hour period. Each treatment was replicated ten times in pairs, such that replicates of each treatment were run simultaneously. Mesocosms were kept in the dark, and red light-illuminated video surveillance was used to observe the first hour of each trial. We recorded the number of *M*. *mercenaria* consumed after 24 hours. Total *M*. *mercenaria* consumption after 24 hours was compared between treatments with a Wilcoxon test. This nonparametric test was used because the consumption data was not normally distributed.

### Recently-introduced intermediate predator naïveté to native top predator: intermediate predator behavioral experiment

We then used a third laboratory experiment in the summers of 2012 and 2013 to test the hypothesis that the recently-introduced intermediate predator would have more pronounced naïveté to the top predator than the native or long-established non-native intermediate predators. These experiments compared behavior of the three intermediate predators when experiencing simulated predation risk through olfactory cues from adult *C*. *sapidus* [34]. Experiments were conducted in 10 gallon aerated tanks (32cm × 26cm × 21cm) which contained 12 cm of water (10cm above 2cm layer of gravel substrate). Individuals of the three intermediate predators were exposed to either: 1) a control treatment with a single food item, an open *M*. *mercenaria* (width of ~50-60mm), and an empty opaque container next to the food item, or 2) an experimental treatment containing an adult *C*. *sapidus* caged in an opaque container next to the food item. For each trial, one intermediate predator individual was introduced into an opaque container at the opposite end of the tank to the food source and other opaque container, where it acclimated for 10 minutes with the aeration on to disperse scents [35]. Aerators were then turned off, the intermediate predator was released from its container, and its behavior was observed for 15 minutes. The two treatments were replicated 10 times for each of the three intermediate predators (N = 60). Behavioral observations of each intermediate predator included the time spent 1) finding the food, 2) consuming the food, and 3) being still. Each of these variables was analyzed using an ANOVA with fixed effects of treatment, species and their interaction, as well as the random effect year. *H*. *sanguineus* responses were compared to *C*. *maenas* and *C*. *sapidus* responses using planned comparison tests.

### Consumer pressure on focal intermediate predators: Tethering experiment

To test the hypothesis that more pronounced naïveté in the recently introduced intermediate predator would result in greater risk of predation, we conducted a field experiment comparing predation rates on *H*. *sanguineus*, *C*. *maenas*, and *C*. *sapidus* in summer 2013. We tethered intermediate predators (3–4 individuals/species) to 45.7cm stakes using braided microfilament line and cyanoacrylate adhesive, and we placed each individual in open, soft sediment habitat three meters apart to minimize multiple predation events caused by a single predator. When using this method, the removal of crabs are indicative of predation events and not tether failure [36]. Intermediate predators were tethered for a 24 hour period and mortality of the individuals of each species was assessed and analyzed using a Pearson’s chi square test.

## Results

### Native basal prey naïveté: *M*. *mercenaria* burrowing experiment

**Fig 1:**
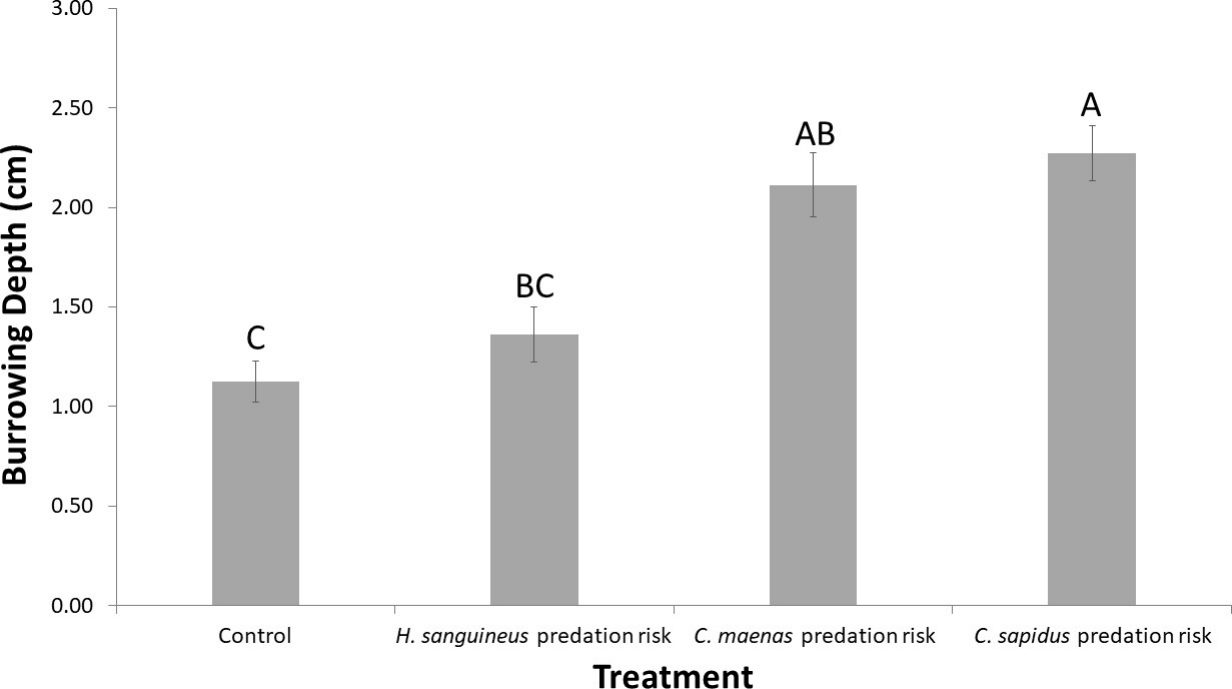
*M*. *mercenaria* burrowing experiment: Mean burrowing depth (cm) over seven-day trials. The four treatments included (1) a control where clams were not exposed to predators, or (2–4) predation risk from *H*. *sanguineus* (recently-introduced non-native species), *C*. *maenas* (long-established non-native species), or juvenile *C*. *sapidus* (native species). Error bars indicate standard error and different letters above bars indicate significant differences among treatments at α = 0.05, as determined using a Tukey’s HSD test.

As hypothesized, the native basal prey demonstrated more pronounced naïveté to the recently-introduced intermediate predator than the long-established or native intermediate predators (Fig 1, Table A in S1 File, R^2^_adj_ = 0.68, F_3/32_ = 6.41, N = 160, treatment p = 0.0016, replicate random effect Wald p = 0.0015). *M*. *mercenaria* did not exhibit anti-predator behavior when exposed to *H*. *sanguineus* chemical cues, and *M*. *mercenaria* burrowing depth was no different in the presence of *H*. *sanguineus* than the control treatment with no predators. The clams burrowed deepest in the presence of *C*. *sapidus*, clearly recognizing the native intermediate predator as a threat. In the presence of *C*. *maenas*, *M*. *mercenaria* burrowed deeper than in the control treatments, suggesting predator recognition, but the depth was not different from that observed in *C*. *sapidus* or *H*. *sanguineus*.

### Foraging benefits to recently-introduced intermediate predator from naïve prey: *H*. *sanguineus* foraging experiment

**Fig 2:**
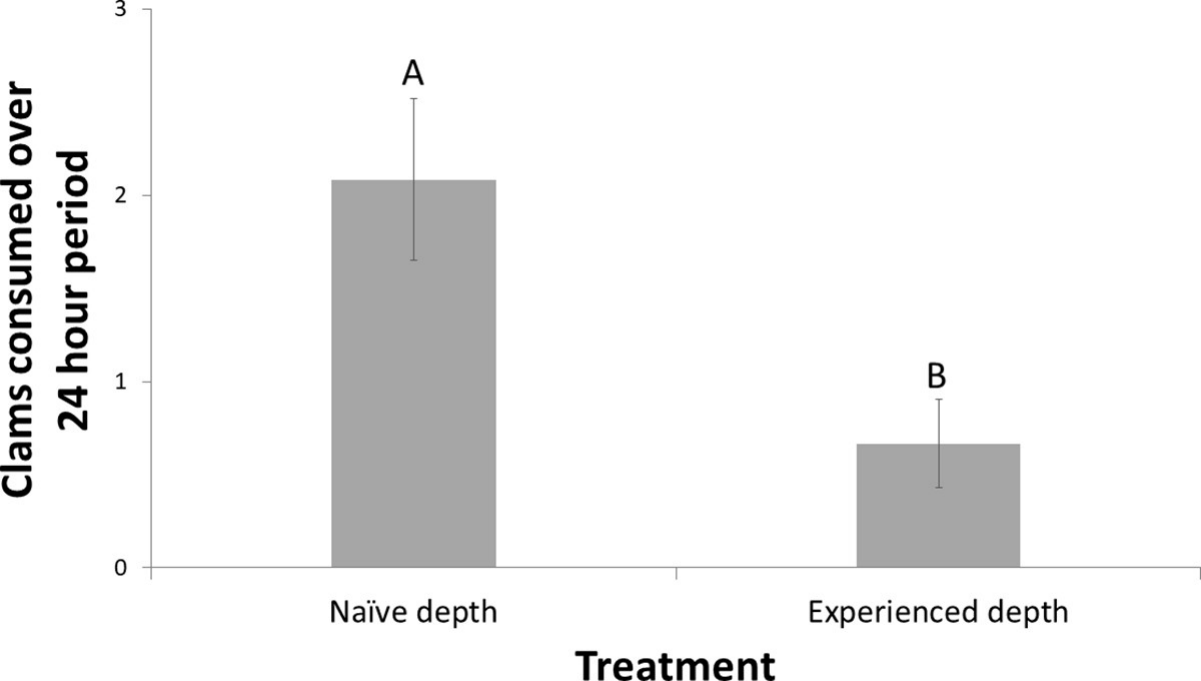
*H*. *sanguineus* foraging success on *M*. *mercenaria* at naïve and experienced burrowing depths (*H*. *sanguineus* foraging experiments): Total *M*. *mercenaria* consumption after 24 hours when burrowed at both a naïve and experienced burial depth. Error bars indicate standard error and letters indicate significant differences in *M*. *mercenaria* consumption between treatments at α = 0.05.

*H*. *sanguineus* gained a direct foraging benefit from naïve *M*. *mercenaria*. Over a 24 hour period, *H*. *sanguineus* consumed twice the number of *M*. *mercenaria* when they were allowed to burrow naturally to their naïve depth in comparison to those placed at their experienced depth (Fig 2, Table B in S1 File, Wilcoxon Test: Z = -2.214 N = 21, p = 0.0268).

### Recently-introduced intermediate predator naïveté to native top predator: Intermediate predator behavioral experiment

**Fig 3:**
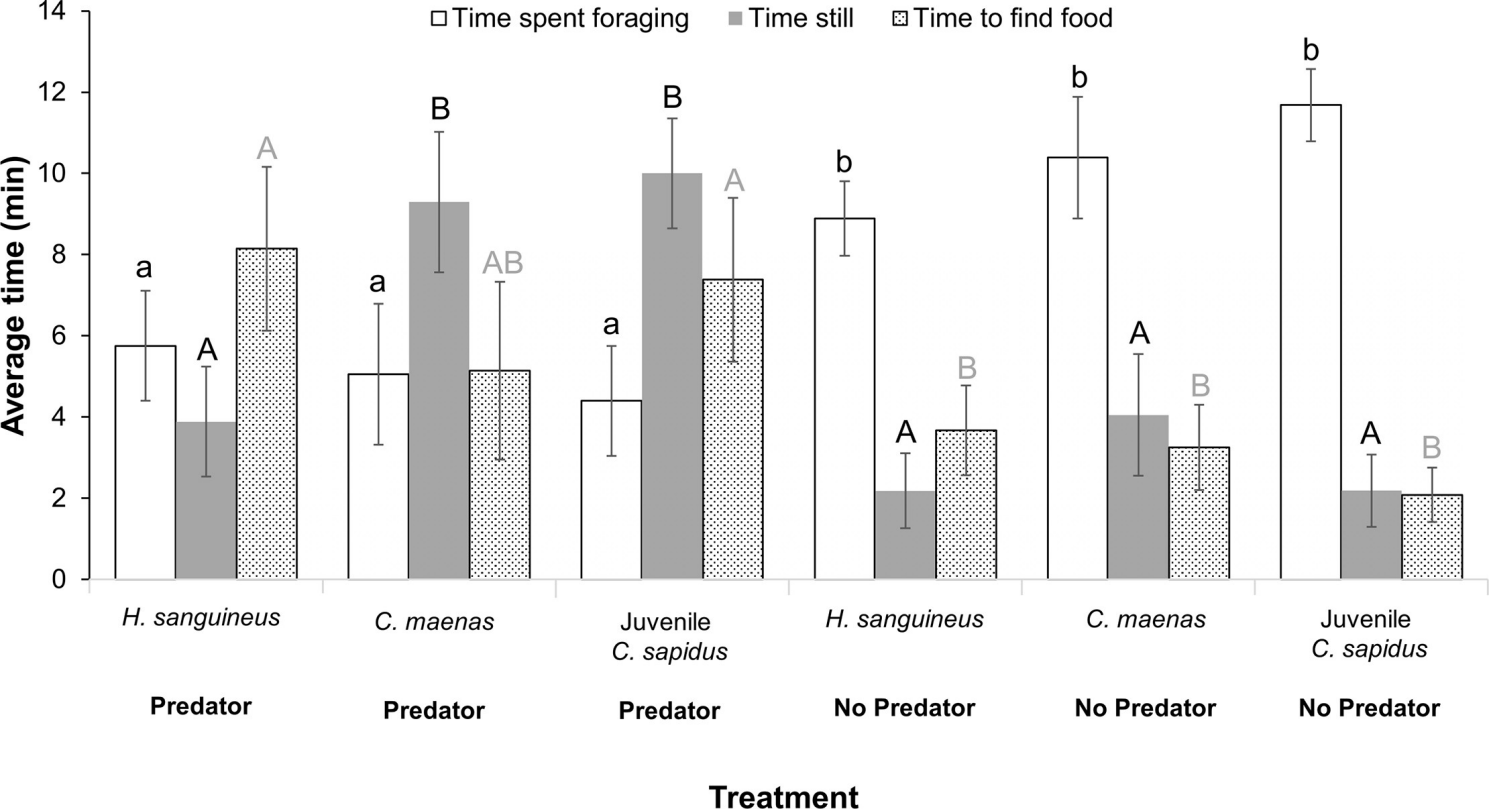
Intermediate predator behavioral comparisons in the presence or absence of a top predator: Time (in minutes) intermediate predators spent foraging (white bars), still (gray bars), and the time to initially detect food (dotted bars) in the presence or absence of a top predator. Error bars indicate standard error and different letters above bars indicate significant differences across treatments at α = 0.05. Lowercase black letters indicate significant differences in time spent foraging across treatments, uppercase black letters indicate significant differences in time still across treatments, and uppercase gray letters indicate significant differences in time to find food across treatments.

The recently-introduced intermediate predator exhibited naïve behavioral responses to the top predator in some but not all aspects of foraging. All intermediate predators were slower to find food in the presence of adult *C*. *sapidus* chemical cues (Fig 3, Table C in S1 File, R^2^_adj_ = 0.06, N = 60, p = 0.07; treatment: p<0.005), but no differences emerged among species (species and treatment×species: p>0.05). Additionally, all intermediate predators reduced feeding time in response to the top predator chemical cues (Fig 3, Table C in S1 File, R^2^_adj_ = 0.20, N = 60, p = 0.003; treatment: p<0.0001; species and treatment×species: p>0.05). The average time spent still in the presence of the top predator, however, depended on the species (Fig 3, Table C in S1 File, R^2^_adj_ = 0.30, N = 60, p<0.0001; treatment: p<0.0001; species: p = 0.02; treatment×species: p = 0.0044, year random effect: Wald p>0.05). All crabs were equally active in the control treatment with no predator (Fig 3, Table C in S1 File), but juvenile *C*. *sapidus* and *C*. *maenas* remained still throughout the majority of the trials in the presence of adult *C*. *sapidus*, indicating predator avoidance (Fig 3, Table C in S1 File). In contrast, *H*. *sanguineus* continued to move in the presence of the top predator (Fig 3, Table C in S1 File). *H*. *sanguineus* therefore reduced its foraging time in the presence of adult *C*. *sapidus*, but still remained active.

### Consumer pressure on focal intermediate predators: Tethering experiment

**Fig 4:**
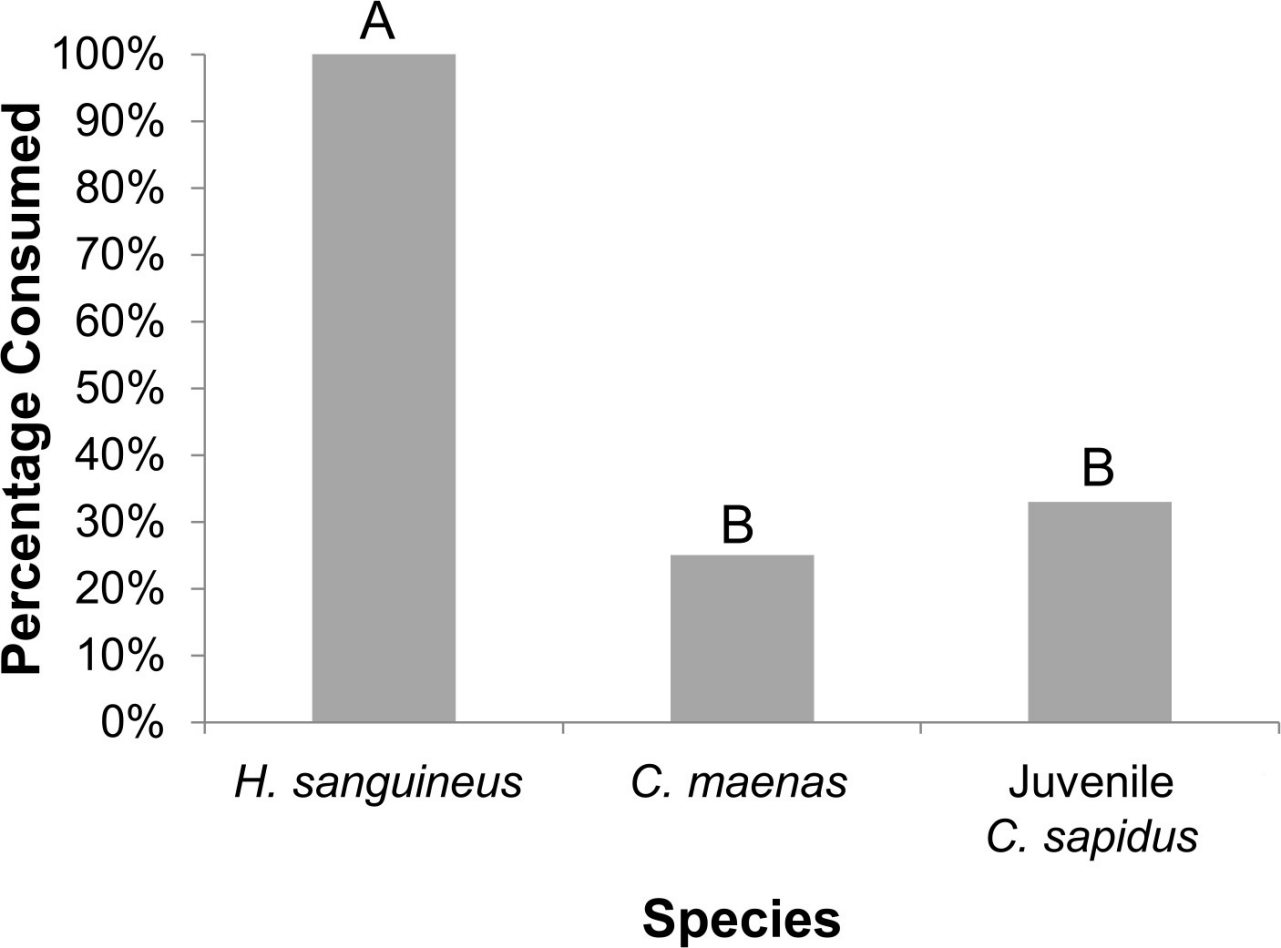
Natural predation in the field on *H*. *sanguineus* compared to *C*. *maenas* and *C*. *sapidus*: Percentage of consumed *H*. *sanguineus*, *C*. *maenas*, and *C*. *sapidus* over a 24 hour period. Different letters indicate significant differences between treatments at α = 0.05.

In the field, a higher predation rate was observed on the recently-introduced intermediate predator in comparison to its long-established and native counterparts (Fig 4, Table D in S1 File, p<0.03, N = 11). During the 24 hour tethering experiment, all *H*. *sanguineus* individuals were consumed, in comparison to the consumption of only one juvenile *C*. *sapidus* and one *C*. *maenas*.

## Discussion

Across two trophic levels, we observed strong naïveté in native basal prey and partial naïveté in the recently-introduced non-native predator. Our native basal prey study species (*M*. *mercenaria*) exhibited a lack of predator recognition [6] in response to the recently-introduced intermediate predator (*H*. *sanguineus*). *H*. *sanguineus* exhibited predator recognition, but an ineffective anti-predator response [6] to a native top predator, potentially explaining the higher predation rate on this species in the field. These results suggest that despite benefitting from basal prey naïveté (resource effect), the recently-introduced intermediate predator’s invasion success may be dampened by its own naïveté (predator effect). Our results highlight that positive and negative effects of naïveté on non-native species may operate simultaneously at different trophic levels, and effect sizes of naïveté may be time-dependent as non-native species become ecologically and evolutionarily integrated into their recipient communities. Future research is needed to understand these effect sizes, as well as how naïveté interacts with other mechanisms, including prey preference and intraguild predation, to ultimately shape predator-prey interactions in invaded communities.

*M*. *mercenaria*, showed a gradient in its behavioral response to predation risk by the three intermediate predators. Burrowing depth, as a measure of predator recognition and avoidance, of these clams was deepest with the native species (*C*. *sapidus*), an intermediate depth with the long-established non-native (*C*. *maenas*), and shallowest with the recently-introduced non-native (*H*. *sanguineus*), with the latter depth being no different than what was observed in a control treatment that lacked a predator. All three predators are functionally similar to one another as they consume bivalve prey via crushing and prying open of shells [27, 37, 38], but naïveté by *M*. *mercenaria* was strongest in response to *H*. *sanguineus* and directly benefited the non-native intermediate predator’s foraging success. Multiple factors could have further influenced the outcome of these results. First, research on non-consumptive predator effects suggests that prey can detect predator chemicals in a concentration-dependent manner that allows them to assess predator proximity and size (either as aggregate biomass or number of individuals [29]). Given the differences in size among our predators in the field and in our experiments, we cannot disentangle the effect of biomass from species identity in our results. Such biomass effects may be ecologically relevant, however, since if *M*. *mercenaria* cannot detect a predation threat from smaller individuals, *H*. *sanguineus’s* smaller adult body size may give it a foraging advantage over the other two species. Secondly, the similarity in burrowing responses to both the long-established *C*. *maenas* and the native *C*. *sapidus* may be due in part to the potential chemical similarities between these species, as they are more closely related to one another than to *H*. *sanguineus* [39] and may produce similar cues which *M*. *mercenaria* recognizes. Recent freshwater data on crustaceans suggest that taxonomic similarity may not influence anti-predator responses [40], but little research has been done on chemical cue similarities among related species in marine systems.

In our study, the weaker response to the recently-introduced non-native predator in comparison to the long-established non-native and native predators, however, is likely related to differences in shared evolutionary history, as predicted by the prey naïveté hypothesis. *H*. *sanguineus* belongs to the Grapsid family of crabs, and in New Jersey, the only native Grapsid crab is *Sesarma reticulatum* [33], which primarily consumes plant and algal matter but not mollusks [41]. *H*. *sanguineus* may therefore produce chemical cues that *M*. *mercenaria* does not recognize since it has no prior exposure to a Grapsid predator, increasing the likelihood of a naïve response. From an evolutionary perspective, population turnover of *M*. *mercenaria* is on the order of 8–10 years in Barnegat Bay and Little Egg Harbor, NJ [42], so only 1–2 generations of clam have been exposed to *H*. *sanguineus* compared to 20–25 to *C*. *maenas*. With several more generations it is possible that the population will alter its burrowing response, as prey often shift to more adaptive anti-predator behavior after the initial period of strong predation [8]. It remains unknown, however, how different *H*. *sanguineus* cues are from those of *C*. *maenas* or *C*. *sapidus*. Cue discrimination abilities are generally acknowledged as a major factor contributing to naïveté [7], so understanding such differences in chemical cues produced by related groups of predators could aid in better predicting when naïveté may occur, and the likelihood of naïve prey being able to eventually adapt to new predators.

*H*. *sanguineus* naïve response to adult *C*. *sapidus* was mixed in that it recognized *C*. *sapidus* as a predator, but demonstrated an ineffective predator avoidance behavior. *H*. *sanguineus* reduced its food consumption in the presence of adult *C*. *sapidus*, which is indicative of predator recognition [43]. Juvenile *C*. *sapidus* and *C*. *maenas*, however, remained more still in the presence of adult *C*. *sapidus*, while *H sanguineus* individuals continued to move. *C*. *sapidus* is an excellent visual predator [44] and this excess movement of *H*. *sanguineus* could increase the top predator’s detection and attack rates on the recently-introduced intermediate predator. Furthermore, *C*. *sapidus* is a fast-moving swimming crab [44] and is likely to outmaneuver smaller, slower prey, such as walking crabs like *H*. *sanguineus*. Remaining still in an attempt to avoid initial detection, therefore, is a more effective predator avoidance strategy, which *H*. *sanguineus* fails to employ. This ineffective predator avoidance behavior may be due to a combination of the short time since introduction and also the lack of a known Portunid (the family to which *C*. *sapidus* belongs) predator in *H*. *sanguineus'* primary native range, the sea of Japan [45]. In contrast, *C*. *maenas*, which did exhibit effective predator avoidance behavior to adult *C*. *sapidus*, has been established in NJ for a longer time than *H*. *sanguineus*, but also has two potential Portunid predators in its native range in the eastern Atlantic, *Necora puber* and *Liocarcinus depurator* [46]. Generation time of *C*. *maenas* is three years [47], thus over 60 generations have been exposed to *C*. *sapidus* predation in NJ. Generation time of *H*. *sanguineus* is only two years [48], but some crustaceans may be able to develop learned predator avoidance behavior within a generation [9]. Thus, *H*. *sanguineus* could develop effective anti-predator behavior for *C*. *sapidus* rapidly. It is possible however that the low densities of *H*. *sanguineus* and *C*. *sapidus* in NJ have kept encounter rates low enough to inhibit the development of a fully experienced anti-predator response.

Another factor contributing to ineffective *H*. *sanguineus* anti-predator response may be that most anti-predator behaviors are adapted for a species’ native environment [49], which for *H*. *sanguineus* is different than the dominant habitat found in our study region. *H*. *sanguineus* native range primarily consists of rocky intertidal habitats [33], and the species’ rapid movements in the presence of *C*. *sapidus* might be useful for quickly seeking shelter from predators in such a habitat. In regions north of the study area, rocky intertidal habitats are quite common, and indeed *H*. *sangineus* has rapidly proliferated there [50]. In NJ and other mid-Atlantic regions where soft sediment habitat is more abundant, *H*. *sanguineus* quick movements are likely ineffective as an anti-predator response. As other invasive species have become successful in habitats different than their native habitats [32], it is possible that *H*. *sanguineus* may eventually adapt to the soft-sediment habitats common to mid-Atlantic environments and alter its behavior to better avoid predators, however the timeline for such a change is unknown.

Perhaps as a consequence of its ineffective anti-predator response, predation on *H*. *sanguineus* in the field was higher than on the long-established *C*. *maenas* or juvenile native *C*. *sapidus*. *H*. *sanguineus* was readily recognized as a resource by higher trophic-level predators in the system. Further, as observed in the presence of *C*. *sapidus*, *H*. *sanguineus* may move in the presence of other visual predators, which could contribute to this higher predation rate. Indeed, various species of fish and other larger omnivorous crabs are abundant in NJ [51] and may also consume *H*. *sanguineus* [52]. It is also important to note that tethering does restrict mobility, and therefore *H*. *sanguineus* may not have been able to exhibit a full anti-predator response. Given how much faster some of the local visual predators are (e.g. *C*. *sapidus* and local fish species) than the walking crab, however, the chance of movement being an effective escape mechanism is unlikely once detected.

Prey preference could also have played a key role in the higher predation rates on *H*. *sanguineus* in the field. Prey preference in marine crustaceans and fish (the likely predators of *H*. *sanguineus* in NJ) is influenced by a variety of factors, including the size relationship between predators and prey (predators often opt for prey with smaller body sizes [52]) and the substrate/prey refuge availability (predators will select prey that have less habitat refuge [52, 53]). While all tethered on the same substrate (soft sediment), the three crabs likely used the substrate differently to avoid predation; in addition to remaining still, both *C*. *maenas* and *C*. *sapidus* are also known to hide by covering themselves with soft sediment (e.g. [54, 55]). *H*. *sanguineus* may not have done this, not knowing how to effectively use the local substrate. Additionally, while the sizes of tethered crabs in our experiment were similar (matched by weight), *H*. *sanguineus* does have a smaller adult body size than the other two species, which may make it available to a broader size class of predators over the course of its life compared to other species [52]. As a result, *H*. *sanguineus*’s ineffective anti-predator response behavior, small adult body size, and potential prey preference of local predators may contribute to its higher predation rates in the field. Regardless, considering the observed rate of loss in our experiment (all individuals consumed), predation pressure may be an important limiting factor for the *H*. *sanguineus* population.

Given that *H*. *sanguineus* benefitted from basal prey naïveté but was also predated upon more heavily, ecological theory suggests that coexistence between *H*. *sanguineus*, *C*. *sapidus*, and *C*. *maenas* may be a possible outcome of this invasion at least in the short term. Intraguild predation systems such as our study system (where species both compete for shared resources and have a predator-prey relationship) are predicted to be stable when the prey (e.g., *H*. *sanguienus*) is superior at exploiting the shared resource, but the predator (e.g., *C*. *sapidus*) gains from consumption of that prey [56, 57]. Further, a third species (e.g., *C*. *maenas*) which may be an inferior competitor on the shared resource (and research does suggest that *H*. *sanguineus* can outcompete *C*. *maenas* for bivalve prey [58, 59]), can be facilitated by the higher predation on the other intraguild predator (e.g. [60]). It is unknown if these species adhere to theoretical expectations, and these dynamics, if operating, would hinge on multi-trophic naïveté that could weaken through time. Nevertheless, it is likely that multi-trophic naïveté affects the novel intraguild predator-prey interactions that occur with invasions.

Although more taxonomic groups need to be studied to determine if our results are broadly applicable to marine systems, our research strongly suggests that a multi-trophic perspective will be critical to understanding how naïveté affects invasion dynamics. Our research demonstrated that naïve native prey behavior likely increases foraging success of a recently-introduced intermediate predator, which could lead to population expansion. When examining these interactions at a higher trophic level, however, this non-native predator also exhibited ineffective anti-predator behavior and suffered strong predation from the native community. This strong predation effect may offset positive resource effects from naïve basal prey and could lead to a stable state of coexistence between the three crab species studied. Investigating the relative importance of these multi-trophic interactions on invasion success either through incorporating behavior into population growth models, examining these interactions at larger spatial scales and/or examining these interactions in the broader context of other interactions (e.g. intraguild predation, competition, facilitation) would inform predictions of where and when naïveté will have the highest impact, either positive or negative, on invasive species establishment.

## Supporting information

S1 File Table A: *M*. *mercenaria* burrowing experiment. Raw data to accompany Fig 1. Table B: *H*. *sanguineus* foraging experiment. Raw data to accompany Fig 2. Table C: Intermediate predator behavioral experiment. Raw data to accompany Fig 3. Table D: Tethering experiment. Raw data to accompany Fig 4.
(XLSX)
